# Effects and outcomes of septostomy in twin-to-twin transfusion syndrome after fetoscopic laser therapy

**DOI:** 10.1186/s12884-019-2555-5

**Published:** 2019-11-01

**Authors:** Wen-Fang Li, An-Shine Chao, Shuenn-Dyh Chang, Po-Jen Cheng, Lan-Yan Yang, Yao-Lung Chang

**Affiliations:** 1 Department of Obstetrics and Gynecology, Chang Gung Memorial Hospital, Chang Gung University College of Medicine, Tao-Yuan, Taiwan; 2 Biostatistics Unit of Clinical Trial Center, Chang Gung Memorial Hospital, Taoyuan, 333 Taiwan; 3 Department of Obstetrics and Gynecology, Chang Gung Memorial Hospital, Linkou Medical Center, 5, Fu-Shin Street, Kweishan, Taoyuan, 333 Taiwan, Republic of China

**Keywords:** Twin-to-twin transfusion syndrome, Septostomy, Laser therapy, Pseudoamniotic band syndrome, Cord entanglement

## Abstract

**Background:**

To evaluate the incidence and outcomes of septostomy in twin-to-twin transfusion syndrome (TTTS) after fetoscopic laser therapy.

**Methods:**

A retrospective analysis of TTTS postlaser septostomy between 2005 and 2018 was performed. Postlaser septostomy was diagnosed using both (1) a free-floating intertwin membrane flap visible on ultrasound examination and (2) the rapid equalization of amniotic fluid maximum vertical pocket in the donor and recipient amniotic sacs observed after laser therapy. Perinatal survival, neonatal brain image anomaly, gestational age at operation and birth, incidence of premature rupture of membranes (PROM) within 3 weeks after operation, pseudoamniotic band syndrome, and cord entanglement were evaluated.

**Results:**

In the 159 TTTS cases included, 12 had postlaser septostomy. Relative to the group without septostomy, the septostomy group had a lower total fetal survival rate (54.2% vs 73.6%, *p* = 0.041), an earlier mean gestational age at delivery (27.8 vs 34.4 weeks, *p* = 0.009), a higher risk of PROMs within 3 weeks after operation (33.3% vs 5.4%, *p* = 0.004), a higher cord entanglement rate (16.7% vs 0%, *p* = 0.005), and a higher brain image anomaly rate (23.0% [3/13] vs 5.0% [11/218], *p* = 0.035). After considering the severe Quintero stages (stage III and IV), postlaser septostomy was the only variable [*p* = 0.003, odds ratio = 5.1] to predict neonatal brain image anomaly. Postlaser septostomy combined with severe Quintero stages could predict PROMs within 3 weeks after laser therapy [*p* = 0.001, odds ratio = 14.1 and *p* = 0.03, odds ratio = 5.4, respectively] and delivery before the gestational age of 28 weeks [*p* = 0.017, odds ratio = 4.5 and *p* = 0.034, odds ratio = 2.3, respectively]. The risk of pseudoamniotic band syndrome was not increased by postlaser septostomy in this case series.

**Conclusions:**

Postlaser septostomy in TTTS was associated with poorer fetal survival and more adverse perinatal outcomes even after considering severe Quintero stages before laser therapy. Efforts should be made to prevent septostomy during laser therapy, and septostomy as the primary method to treat TTTS is not advisable.

## Background

Twin-to-twin transfusion syndrome (TTTS) is defined by a preferential shunting of blood from one twin (donor) to the other twin (recipient) through vascular communications [1], which occurs in approximately 9% of monochorionic diamniotic twin pregnancies [2]. Fetoscopic laser therapy is recognized as the first-line therapy for TTTS diagnosed before 26 weeks of gestation [3–5]. Septostomy designates the perforation of the membrane separating the twins that can occur after laser therapy for TTTS [1, 6–9]. Septostomy after laser therapy for TTTS can be caused by the perforation of the donor’s collapsed membrane at the trocar insertion site, laser photocoagulation through the dividing membrane, or mechanical rupture of the membrane during the operation by the laser fiber tip or trocar tip. The reported incidence of septostomy after laser therapy for TTTS ranges from 1.6 to 25.0% [1, 6, 7, 9, 10]. Postlaser septostomy is associated with higher risks of early delivery [1, 7, 9], intrauterine fetal demise (IUFD) [7], and cerebral injury in the surviving fetus [9].

In this study, we included TTTS cases with and without septostomy after laser therapy before the gestational age of 26 weeks to assess the incidence of this event and its associated outcomes.

## Methods

This study performed at the Chang Gung Memorial Hospital, Taoyuan, Taiwan was approved by the Institutional Review Board of the Chang Gung Medical Foundation (IRB # 201801353B0). Only two laser therapy centers for TTTS currently serve the 23.7 million residents of Taiwan. Ours is the larger laser therapy center and has handled more than 95% of cases. All laser operations for TTTS were performed by a single operator (YL Chang). The diagnosis of TTTS was based on ultrasound findings as defined by Quintero et al. [11]. From October 2005 to November 2018, 159 TTTS cases diagnosed before the gestational age of 26 weeks and who received fetoscopic laser therapy at our hospital were included in this study. The procedure of laser therapy for TTTS in our hospital has been described in a previous study [12].

The preoperative characteristics, including maternal age, Quintero staging, and gestational age at operation, were assessed and documented in TTTS cases with and without septostomy after laser therapy. Patients with TTTS were followed up with serial ultrasound 1 day before, and every day after laser therapy if the patient was admitted. Quintero stage III and IV are considered severe Quintero stages. Septostomy was diagnosed based on both (1) a free-floating intertwin membrane flap visible on ultrasound examination and (2) the rapid equalization of amniotic fluid maximum vertical pocket in the donor and recipient amniotic sacs [1].

The outcomes of fetoscopic laser therapy for TTTS, including fetal survival (defined as living more than 30 days after delivery), gestational age at delivery, premature rupture of membranes (PROM) within 3 weeks after the operation, interval between laser therapy and delivery, pseudoamniotic band syndrome, and cord entanglement, were compared between the two groups of TTTS. All live neonates received cranial ultrasound examination within 1 month after delivery. If two or more cranial ultrasound tests were performed, the last examination result was selected. Mild cerebral image anomaly was identified by lesions detected through cranial ultrasound scans with the presence of at least one of the following: intraventricular hemorrhage (IVH) grade I and II, lenticulostriate vasculopathy, and subependymal pseudocysts. Severe cerebral image anomaly was based on the presentation of one of the following signs: IVH grade III or grade IV, cystic periventricular leukomalacia grade II or more, porencephalic cysts, and ventricular dilatation. Ventricular dilatation was diagnosed when the width of the unilateral or both lateral ventricles exceeded the 97th percentile [12].

Statistical analysis was conducted using SPSS (version 11.0 for Windows; SPSS Inc., Chicago, IL, USA). Data are expressed as mean ± standard deviation, median (min, max), and frequency (%), as and when appropriate. Qualitative data were compared using the χ^2^ test or Fisher exact test. Continuous variables were tested for normality. Student’s t-test or Mann–Whitney *U* test was used to compare the continuous variables between groups. A *p*-value of less than 0.05 was considered statistically significant. Logistic regression (forward condition) was selected to determine the predisposing factors for poor fetal outcomes, including brain image anomaly, PROM within 3 weeks after laser therapy, and delivery before the gestational age of 28 weeks. Septostomy after laser therapy, maximum vertical pocket (MVP) of the recipient, gestational age at laser therapy, and severe Quintero stage (i.e., stage III and IV) were picked as the variables to enter the mode when the *p*-value was less than 0.05 and removed when the probability value was more than 0.1. The odds ratios of the variables were expressed as odds (95% confidence interval).

## Results

Among the 159 postlaser therapy TTTS cases that met the inclusion criteria during the study period, 7.5% (12/159) patients had septostomy. In 58.3% (7/12) of the cases, the operator was already aware of the perforation during the procedure, and in 41.7% (5/12), septostomy was observed the day after the operation through ultrasound examination. Among the patients, 17.0% (27/159) were at the Quintero stage I, 34.6% (55/159) at the stage II, 34.0% (54 /159) at the stage III, and 14.5% (23 /159) at the stage IV. Of the 12 patients with postlaser septostomy, 7.4% (two cases) were at the Quintero stage I, 10.9% (six cases) were at the stage II, 3.7% (two cases) were at the stage III, and 8.7% (two cases) were at the stage IV. There was no significant difference in the incidence of postlaser septostomy according to the Quintero stage (*p* = 0.56, chi-squared test). The median gestational age at operation in all TTTS cases was 20.7(16.0, 26.0) weeks. 7.5% (12 patients) experienced PROM within 3 weeks after the operation. The median gestational age at delivery was 34.0(19.9, 40.0) weeks, the total fetal survival rate was 72.0% (229/318), the single survival rate was 86.7% (138/159), and the dual survival rate was 57.2% (91/159).

The two groups of TTTS with and without septostomy after laser therapy did not differ significantly in terms of the maternal age and gestational age at operation. TTTS cases with postlaser septostomy exhibited an earlier delivery [27.8 (19.9, 37.3) vs 34.4 (20.3, 40.0) weeks, respectively, *p* = 0.011] and a shorter interval between the operation and delivery [24 (8, 115) vs 88 (1, 159) days, respectively, *p* = 0.002]. The survival rate for at least one of the twins (75.0% vs 89.1%, respectively, *p* = 0.047) and total fetal survival rate (54.2% vs 73.6%, respectively, *p* = 0.041) after laser therapy were lower in the TTTS group with postlaser septostomy than in the group without. The risk of PROM within 30 days after laser therapy (33.3% vs 5.4%, respectively, *p* = 0.004) was also higher in the TTTS group with postlaser septostomy than in the group without (Table 1).

**Table 1:** Characteristics of the patients with twin-to-twin transfusion syndrome patients with and without septostomy after laser therapy

	With post laser septostomy (*N* = 12)	Without post laser septostomy (*N* = 147)	*P* value
Maternal age at operation (year-old)	29.6 (24.9, 42.2)	31.3 (23.4, 43.4)	0.43^a^
Gestational age at operation (weeks)	21.1 (18, 25)	20.7 (16, 26)	0.19^a^
Gestational age at delivery (week)	27.8 (19.9, 37.3)	34.4 (20.3, 40.0)	0.011^a^
PROMs within 3 weeks after operation ((%)number)	33.3% (4/12)	5.4% (8/147)	0.004^c^
Interval from operation to delivery (days)	24 (8115)	88 (1, 159)	0.002^a^
Cord entanglement ((%)number)	16.7% (2/12)	0% (0/147)	0.005^c^
Amniotic band syndrome	0% (0/12)	1.4% (2/147)	1.0^c^
Severe brain image anomaly	7.6% (1/13)	1.4% (3/218)	0.21^c^
Mild brain image anomaly	15.4% (2/13)	4.1% (8/218)	0.10^c^
Total brain image anomaly	23.0% (3/13)	5.0% (11/218)	0.035^c^
Dual fetal survival ((%)number)	41.7% (5/12)	59.2% (87/147)	0.24^b^
At least one fetal survival((%)number)	75.0% (8/12)	89.1% (131/147)	0.047^c^
Total fetal survival ((%)number)	54.2% (13/24)	73.6% (218/296)	0.041^b^

Data are expressed as median (min, max) and percentage (frequencies)

Mild cerebral image anomaly: defined by lesions detected in cranial ultrasound scans with the presence of at least one of the following: intraventricular hemorrhage (IVH) grade I and II, lenticulostriate vasculopathy, and subependymal pseudocysts

Severe cerebral image anomaly: defined by the presentation of one of the following signs: IVH grade III or grade IV, cystic periventricular leukomalacia grade II or more, porencephalic cysts, and ventricular dilatation. Wentricular dilatation was diagnosed when the width of the unilateral or of both lateral ventricles exceeded the 97th percentile

*PROMs* Premature rupture of membranes

^a^Mann–Whitney *U* test

^b^chi-squared test

^c^Fisher’s exact test

The incidence of postlaser septostomy was not significantly associated with Quintero stage (Table 2). That is, patients with TTTS at a severe Quintero stage did not face an increased risk of postlaser septostomy.

**Table 2:** Incidence of postlaser septostomy in TTTS was not significantly different among the Quintero stages

	Stage I	Stage II	Stage III	Stage IV	*P* value
Septostomy %(number)	7.4% (2/25)	10.9% (6/49)	3.7% (2/52)	8.7% (2/21)	0.56

*P* value was generated using the chi-squared test

Logistic regression analyses were conducted to predict the poor fetal outcomes, including brain image anomaly, PROM within 3 weeks after the operation, and delivery before the gestational age of 28 weeks. Septostomy after laser therapy, MVP of the recipient, gestational age at laser therapy, and severe Quintero stage (i.e., stage III and IV) were selected as variables. Postlaser septostomy was the only variable [*p* = 0.03, odds ratio = 5.1 (1.18–22.2)] predicting neonatal brain image anomaly. A combination of postlaser septostomy and severe Quintero stage predicted PROM within 3 weeks after laser therapy [*p* = 0.001, odds ratio = 14.1 (2.8–70.1) and *p* = 0.03, odds ratio = 5.4 (1.2–25.0), respectively] and delivery before the gestational age of 28 weeks [*p* = 0.017, odds ratio = 4.5 (1.3–15.8) and *p* = 0.034, odds ratio = 2.3 (1.1–5.1), respectively] (Table 3).

**Table 3:** Logistic regression to predict the poor neonatal outcomes including brain image anomaly, PROM within 3 weeks after laser operation, and delivery before the gestational age of 28 weeks in TTTS postlaser therapy

Observed poor outcomes	Variable 1(septostomy)	Variable 2 (severe Quintero stage)
Neonatal cerebral image anomaly	(*p* = 0.03, Odds ratio = 5.1 (1.18–22.2))	Not significant
PROMs within three weeks after laser operation	(*p* = 0.001,Odds ratio = 14.1 (2.8–70.1))	(*p* = 0.03, Odds ratio = 5.4 (1.2–25.0))
Delivery before gestational age of 28 weeks	(*p* = 0.017, Odds ratio = 4.5 (1.3–15.8))	(*p* = 0.034, Odds ratio = 2.3 (1.1–5.1))

The odds ratios of the variables were expressed as odds ratio (95% confidence interval)

Severe Quintero stages included Quintero stage III and stage IV

*PROMs* Premature rupture of membranes

*TTTS* Twin-to-twin transfusion syndrome

Two cases presented with cord entanglement in the TTTS group with postlaser septostomy at delivery. One presented with dual IUFD 7 days after the operation, and cord entanglement was observed on ultrasound examination and confirmed after delivery. In the other case, the donor presented with IUFD 7 days after the operation, and the recipient twin presented with fetal distress and underwent emergent cesarean section at the gestational age of 31 weeks, when cord entanglement was observed during the operation. Because there was no case of cord entanglement in the group without postlaser septostomy, the risk of cord entanglement was significantly higher in the group with postlaser septostomy (16.7% vs 0%, respectively, *p* = 0.005).

Two cases with pseudoamniotic band syndrome at delivery were observed. In one case, only one of the twins survived (the recipient) and presented with an amputation of the right big toe. In the other case, both twins survived but the recipient twin presented with a contraction of the right lower leg. Both cases were part of the group without postlaser septostomy. Therefore, the risk of pseudoamniotic band syndrome was not significantly different between the TTTS groups with and without postlaser septostomy (0% vs 1.4%, respectively, *p* = 1.0).

In the 296 live babies that survived for more than 30 days, 4.7%(14 cases) were diagnosed as having brain injury based on cranial ultrasound. Among them, 1.4%(four cases) cases had severe brain anomaly. The total brain anomaly rate was higher in the TTTS group with postlaser septostomy than in the group without [23.0% (3/13) vs 5.0% (11/218), respectively, *p* = 0.035]. However, the severe brain image anomaly rate was not significantly different between the two groups of TTTS (Table 1).

## Discussion

This study revealed the incidence of septostomy in TTTS after laser therapy was 7.5%.Its occurrence was associated with an increased risk of adverse perinatal outcomes such as a lower fetal survival rate, higher risks for fetal brain image anomaly, higher incidence of PROM and prematurity, and increased risks of cord entanglement. The incidence of major brain image anomaly and pseudoamniotic band syndrome did not increase in TTTS with postlaser septostomy in the case series.

Quintero stage has been recognized as correlated with perinatal outcomes [13, 14]. Severe Quintero stages (stage III and IV) have also been associated with lower fetal survival in TTTS treated by laser therapy [15]. To clarify whether severe Quintero stage alone, septostomy alone, or these two factors combined resulted in poor fetal outcomes, we performed multivariate logistic regression analyses to predict the poor fetal outcomes including brain image anomaly, PROM within 3 weeks after laser therapy, and delivery before 28 weeks of gestational age. Based on the results, we hypothesized that, even when considering severe Quintero stage before laser therapy in TTTS, postlaser septostomy remained a significant variable predicting neonatal brain image anomaly, PROM within 3 weeks after the operation, and delivery before the gestational age of 28 weeks.

The incidence of postlaser septostomy has been reported as high as 25.0% in a new laser therapy center for TTTS [10]. In other case series, the incidence ranged from 1.6 to 20.0% [1, 6, 7, 9]. According to our experience, the causes of postlaser septostomy may be the perforation of the donor’s collapsed membrane at the trocar insertion site, laser photocoagulation through the dividing membrane, or mechanical rupture of the membrane during the operation by the laser fiber tip or trocar tip. In some TTTS pregnancies with mainly anterior placenta, septostomy may be inevitable because of the limited available site for trocar insertion. The risk of postlaser septostomy in TTTS caused by laser photocoagulation through the dividing membrane especially with blood clot in the donor sac or mechanical rupture of the membrane by the instrument tip could be reduced by more experience of laser operation. A reduced septostomy during laser therapy for TTTS would certainly improve the perinatal outcomes. The increased prematurity in the neonates associated with postlaser septostomy in our patients with TTTS was mainly due to the shorter interval between operation and delivery, given that the gestational age at operation was not significantly different between TTTS with and without postlaser septostomy.

Severe cerebral injury has been reported to significantly increase in the postlaser septostomy group [9]. In our limited data, the total brain image anomaly rate was higher in the TTTS group with postlaser septostomy, but the severe brain image anomaly rate was not significantly different between the survivors of the two groups of TTTS.

16.6% (two cases) of the 12 patients with TTTS with septostomy after laser therapy faced cord entanglement, which was confirmed at delivery. Previous reports have indicated that the risk of cord entanglement in postlaser septostomy TTTS is 12.0% [9] and 26.9% [1] at birth. In one of the two cases, the two twins had IUFD, and in the other case, one twin survived and faced fetal distress. Therefore, the risk of adverse perinatal outcomes in patients with TTTS with postlaser septostomy and cord entanglement is extremely high.

Interestingly, two cases of pseudoamniotic band syndrome were detected in this case series, which was similar to a previous report. They were both from the group without postlaser septostomy [9]. In both cases, the affected parties were the recipient twins. The postlaser fetal middle cerebral artery peak systolic velocities were not significantly discordant between the two fetuses in a TTTS pair in both cases, so twin anemia-polycythemia sequence was unlikely. Hypercoagulation status with a vascular occlusion event in the recipient twin may be associated with limb anomaly as suspected.

The strength of this study was that it was based on data from a single center with consecutive cases, and all the laser operations were performed by a single operator (YLC). Operations were performed following the same procedure and using the same instruments. Therefore, technique and instrument bias can be excluded. The limitations of this study included the small case number and the use of cranial ultrasound to detect brain image anomaly. The increasing incidence of major brain image anomaly demonstrated by a previous study [9] could not be concluded by this study. Given that septostomy after laser therapy for TTTS was associated with a lower fetal survival and more adverse outcomes based on our study, we concluded similarly to other reports that septostomy as a primary method to treat TTTS is not advisable [1, 7, 9].

Fetoscopic laser therapy is universally recognized as the first-line therapy for stage II to IV TTTS diagnosed before 26 weeks of gestation [3], although such use for stage I TTTS is under debate [4]. The survival rate for stage I TTTS was 94.4% for at least one twin and 77.7% for both twins [15]. The incidence of postlaser septostomy did not differ significantly between the Quintero stages in our series. The North American Fetal Therapy Network reported that 60.0% of stage I TTTS that had been managed conservatively progressed to advanced stages, and only fetoscopic laser surgery but not amnioreduction was protective against double fetal loss or very preterm delivery before 26 weeks [4]. Therefore, as demonstrated by our series, offering laser therapy to stage I TTTS patients before a gestational age of 26 weeks is reasonable, although this might involve risks such as postlaser septostomy.

Because postlaser septostomy is associated with poor fetal outcomes, the careful selection of the trocar insertion site for TTTS laser therapy is imperative for preventing septostomy during trocar insertion. Gentle scope and laser fiber manipulation during operation is crucial to prevent mechanical trauma to the intertwin membrane. No differences in the incidence of cord entanglement or fetal survival rate were observed between TTTS patients with postlaser septostomy managed through outpatient surveillance and those with postlaser septostomy managed through inpatient surveillance. However, the gestational age at delivery was earlier in cases with inpatient management [1]. Thus, the optimal management method for TTTS with postlaser septostomy is still unclear.

## Conclusions

The risk of postlaser septostomy was 7.5% in patients with TTTS treated with laser therapy in our study, and its occurrence was associated with an increased risk of adverse perinatal outcomes. Based on our data, we conclude that septostomy as a primary method to treat TTTS is not advisable, and effort should be made to prevent septostomy during laser therapy for TTTS.

## Data Availability

The datasets obtained and/or analyzed during the current study are available from the corresponding author on reasonable request.

## Abbreviations

IUFD: Intrauterine fetal demise
IVH: Intraventricular hemorrhage
MCA-PV: Middle cerebral artery-peak velocity
MRI: Magnetic resonance imaging
PROM: Premature rupture of membranes
PVL: Periventriculoleukomalacia
TAPS: Twin anemia-polycythemia sequency
TTTS: Twin-to-twin transfusion syndrome
